# Combination of High‐Fat Diet and Chronic Unpredictable Stress Synergistically Induces Osteoarthritis‐Like Changes in Temporomandibular Joints in Rats

**DOI:** 10.1155/mi/1164213

**Published:** 2026-07-18

**Authors:** Xi Wang, Ting Hao, Ye-Hua Gan

**Affiliations:** ^1^ Central Laboratory, Peking University School and Hospital of Stomatology, Beijing, China, bjmu.edu.cn; ^2^ Center for TMD and Orofacial Pain, Peking University School and Hospital of Stomatology, Beijing, China, bjmu.edu.cn; ^3^ Peking University School and Hospital of Stomatology, National Center of Stomatology, National Clinical Research Center for Oral Diseases, National Engineering Research Center of Oral Biomaterials and Digital Medical Devices, Beijing Key Laboratory of Digital Stomatology and Research Center of Engineering and Technology for Computerized Dentistry Ministry of Health, Beijing, China, bjmu.edu.cn; ^4^ Center of Stomatology, Beijing Tsinghua Changgung Hospital, School of Clinical Medicine, Tsinghua Medicine, Tsinghua University, Beijing, China, tsinghua.edu.cn

## Abstract

**Objectives:**

High‐fat diet (HFD) and chronic unpredictable stress (CUS) are potential risk factors for temporomandibular joint osteoarthritis (TMJOA). This study aimed to investigate whether the combination of HFD and CUS synergistically induces pathological changes in the temporomandibular joints (TMJs) and to explore the underlying molecular mechanisms.

**Methods:**

Male Sprague‐Dawley rats were assigned to four groups as follows normal diet (ND), HFD, ND with CUS (ND/CUS), and HFD with CUS (HFD/CUS), and were treated with HFD, CUS, or both accordingly. TMJs were harvested after 5 or 10 weeks of HFD, CUS, or combined HFD/CUS treatment. An additional group was used to evaluate whether the TLR4 inhibitor TAK242 could attenuate HFD/CUS‐induced TMJOA‐like changes. Serum proteins or lipids were measured by enzyme‐linked immunosorbent assay (ELISA) or biochemical analysis. Pathological changes were evaluated using microcomputed tomography (micro‐CT) for subchondral bone morphometry, histology with Mankin scoring for cartilage degradation, and TUNEL assays for chondrocyte apoptosis. The expression levels of TLR4, NF‐κB p65, and IL‐1β in condylar cartilage were assessed by immunofluorescence.

**Results:**

The HFD/CUS group showed serum levels of total cholesterol (TC), triglycerides, and oxidized LDL (ox‐LDL) comparable to those in the HFD group, and both groups had significantly higher levels than the ND and ND/CUS groups. Simultaneously, the HFD/CUS group also exhibited the earliest and most severe TMJOA‐like pathological changes and highest Mankin score, including cartilage degradation, subchondral bone resorption, and increased chondrocyte apoptosis as early as 5 weeks posttreatment. The ND/CUS and HFD groups only showed slight degenerative changes at 5 weeks posttreatment and obvious TMJOA‐like changes at 10 weeks posttreatment. The HFD/CUS group also showed a higher number of TLR4‐positive cells, NF‐κB p65‐nuclear‐positive cells, and IL‐1β‐positive cells in the condylar chondrocytes than those in the ND/CUS and HFD groups at 5 weeks posttreatment. TAK242 significantly alleviated the cartilage degradation, subchondral bone destruction, and chondrocyte apoptosis in the HFD/CUS group.

**Conclusion:**

HFD and CUS could synergistically induce TMJOA‐like changes, potentially by activating the TLR4/NF‐κB/IL‐1β inflammatory signaling pathway. Our findings suggest an important interplay between metabolic and psychological factors in the pathogenesis of TMJOA.

## 1. Introduction

Temporomandibular joint osteoarthritis (TMJOA) is a common degenerative joint disease characterized by progressive cartilage degradation, subchondral bone remodeling, and synovial inflammation [1]. The etiology of TMJOA is usually thought to be multifactorial and remains to be fully understood. A biopsychosocial theory of interplay among biological, psychosocial, and social factors is presumably often employed to explain the pathogenesis of TMJOA [2]. However, current TMJOA animal models predominantly rely on intra‐articular drug administration [3], the induction of malocclusion [4], or partial disk removal [5], neither of which could well mimic the potential clinical etiologies of TMJOA. So far, there still lacks experimental evidence to demonstrate the interplay between the psychosocial factor, such as chronic psychosocial stress and the social factor, such as lifestyle, in the pathogenesis of TMJOA.

Epidemiological evidence indicates that chronic psychological stress, a common feature of modern society, often coexists with TMJOA [6]. Psychological stress disrupts neuroendocrine homeostasis by activating the hypothalamic–pituitary‐adrenal (HPA) axis and the sympathetic nervous system [7]. This activation promotes the release of proinflammatory cytokines (e.g., IL‐1β, TNF‐α) and simultaneously impairs the anti‐inflammatory feedback regulation of endogenous glucocorticoids [8]. Besides, stress‐related behaviors may also exacerbate mechanical loading on the temporomandibular joint (TMJ) [9], while neurogenic inflammation sensitizes peripheral nociceptors, contributing to persistent pain and tissue damage [10]. These studies suggest that psychological stress is not merely a psychosomatic mediator but may also potentially serve as a systemic driver of TMJOA. A few studies have pioneered the chronic unpredictable stress (CUS) models in rodents to investigate the pathological changes and the underlying molecular mechanisms in TMJs. CUS can induce a progressive, time‐dependent deterioration of condylar cartilage, mechanistically linked to the elevated secretion of inflammatory cytokines and degradation of the extracellular matrix (ECM) [11].

A high‐fat diet (HFD), which can lead to dysregulation of serum lipid metabolism, is increasingly recognized for its capacity to induce chronic low‐grade systemic inflammation [12, 13]. HFD‐induced inflammation, sometimes referred to as meta‐inflammation (metabolic inflammation accompanying metabolic diseases), has been implicated in the pathogenesis of various chronic diseases [14], including osteoarthritis of limb joints [15, 16]. Previous studies also suggested a potential role of HFD in the pathogenesis of TMJOA in rats [17–19].

While various established animal models share a common endpoint of TMJOA‐like degeneration, they exhibit considerable heterogeneity in disease onset and progression. For instance, chemically induced models, such as the monosodium iodoacetate (MIA) injection, trigger rapid and severe cartilage destruction as early as day 3 [20]. Surgical interventions like TMJ discectomy induce condylar swelling and hyperplasia at 4 weeks, progressing to horizontal splitting and chondrocyte clustering at 8 weeks and extensive fibrillation by 12 weeks [21]. Similarly, force‐induced models like the unilateral anterior crossbite (UAC) lead to chondrocyte disorganization at 2 weeks, disrupted layer demarcation at 4 weeks, and progressive proteoglycan loss from 8 to 20 weeks. In contrast to these localized, rapid‐onset models, systemic risk factors such as HFD or CUS may initiate a slowly evolving pathological process, in which early alterations at 5 weeks are characterized by a disrupted cellular organization within the condyle.

Recent studies have also revealed a bidirectional relationship between HFD and psychological stress. HFD can exacerbate the physiological effects of stress, such as heightened inflammatory responses and increased oxidative stress [22]. On the other hand, psychological stress can also alter dietary preferences, promoting HFD consumption and further aggravating metabolic dysregulation [23]. Therefore, based on the frequent co‐occurrence of HFD and chronic psychological stress in modern society, we hypothesized that the combination of the two risk factors could lead to a synergistic effect on the development of TMJOA.

The interplay between psychological stress and metabolic dysregulation may activate crosstalk between the HPA axis and Toll‐like receptor 4 (TLR4) signaling [24–26]. TLR4, a key pattern recognition receptor in innate immunity, is upregulated under chronic stress conditions due to glucocorticoid resistance and sympathetic signaling [27]. This upregulation heightens the joint microenvironment responsiveness to endogenous danger signals and exogenous ligands [28, 29]. Activation of TLR4 also triggers NF‐κB nuclear translocation, leading to the expression of proinflammatory mediators, such as IL‐1β and COX‐2, which, in turn, exacerbates cartilage catabolism and synovitis [12, 30–32]. In addition, ox‐LDL can promote TLR4 expression and nuclear translocation of NF‐κB through TLR4 in vascular smooth muscle cells [33]. Accordingly, it is necessary to examine whether TLR4 is functionally involved in the interplay of CUS and HFD in the pathogenesis of TMJOA.

Therefore, this study aimed to investigate whether the combination of HFD and CUS could synergistically induce pathological changes in TMJs in rats and, mechanistically, whether the TLR4/NF‐κB/IL‐1β signaling pathway could be involved.

## 2. Materials and Methods

### 2.1. Animals

The Peking University Health Science Center Animal Ethics Committee approved this study (Approval ID: DLASBD 0002), which complied with the ARRIVE guidelines. 5‐week‐old male Sprague–Dawley rats weighing 200 ± 20 g (Beijing Sibeifu Biotechnology Co., Ltd., Beijing, China) were housed and acclimated in a specific pathogen‐free facility at the School of Stomatology, Peking University, under a controlled temperature and a 12‐h light/dark cycle. The rats (*n* = 6 each) were then randomly assigned to receive a normal diet (ND), a HFD, a ND with CUS (ND/CUS), or a HFD with CUS (HFD/CUS) for 5 and 10 weeks. Six rats from each group were then euthanized for microcomputed tomography (micro‐CT) TMJ imaging and histopathological evaluation.

### 2.2. HFD and CUS Protocol

HFD was provided to the rats in the HFD and HFD/CUS groups from Fanbo Biotechnology. The diet consisted of 45% kcal from fat, 35% from carbohydrates, and 20% from protein and was maintained until the end of the experimental period. The CUS protocol was adapted from Willner et al. [34]. Rats were exposed to eight different stressors: noise exposure (95–100 dB, 2 h), damp sawdust bedding (24 h), empty cage (24 h), restraint stress (3 h), forced swimming in 25°C water (20 min), inversion of the light/dark cycle (12 h), cold water immersion at 4°C (7 min), and cage tilting at a 45° angle (5 h). One stressor was randomly applied each day, with no repetition within the same week to prevent habituation. For the inhibitory experiment, rats were treated for 5 weeks under the same HFD/CUS protocol. TAK242 was intraarticularly injected into bilateral TMJs at a concentration of 10 μg/mL, 50 μL per joint, twice per week from the beginning of the HFD/CUS protocol. Rats in the ND and HFD/CUS control groups received equal volumes of saline injections. TMJ samples were collected at 5 weeks for micro‐CT, histology, immunofluorescence, and TUNEL analyses.

### 2.3. Enzyme‐Linked Immunosorbent Assay (ELISA) and Biochemistry Analysis

Blood samples were collected from the vein (*n* = 6 per group per time point). Serum levels of IL‐1β, corticosterone (CORT), adrenocorticotropic hormone (ACTH), and oxidized LDL (ox‐LDL) were measured using ELISA kits (MultiSciences Biotech Co., Ltd. and Elabscience) according to the manufacturers’ instructions. Briefly, serum samples were added to wells precoated with specific antibodies, incubated at 37°C for 30 min, and washed thoroughly. HRP‐conjugated secondary antibodies were added, followed by incubation at 37°C for another 30 min. After washing, a chromogenic substrate was added and incubated for 10 min at 37°C. Absorbance was measured at 450 nm using a microplate reader (Revvity VICTOR, Nivo). Serum total cholesterol (TC) and triglycerides were analyzed with biochemistry using biomedical assay kits (Nanjing Jiancheng, Nanjing, China).

### 2.4. Micro‐CT Analysis

TMJs (*n* = 6 per group per time point) were dissected and preserved in phosphate‐buffered saline (PBS) before scanning with a micro‐CT system (Inveon, Siemens) at 80 kV, 500 μA, and a 9.24 μm pixel size. Bone structure parameters, including bone volume/tissue volume (BV/TV) and bone mineral density (BMD), were analyzed using the manufacturer’s software, Inveon Research Workplace. The entire condyle was segmented across 20 sagittal slices, and the mean BV/TV and BMD values were calculated for each group.

### 2.5. Histological Analysis

TMJ tissue sections were stained with hematoxylin and eosin (HE) for general evaluation and toluidine blue (TB) for cartilage integrity analysis. TMJOA severity was quantified using a modified Mankin scoring system [35] by three blinded researchers. All analyses were performed in a blinded manner to minimize bias.

### 2.6. Immunofluorescence Staining

Paraffin‐embedded sections were deparaffinized in xylene and rehydrated through graded ethanol. Antigen retrieval was performed in citrate buffer (10 mM, pH 6.0) at 100°C for 2 min. Sections were blocked with 3% hydrogen peroxide and incubated with 10% normal serum for 30 min. Primary antibodies, including anti‐IL‐1β (Bioss, bs‐0812), anti‐p65 (HUABIO, ET1603‐12), and anti‐TLR4 (Proteintech, 66350‐1‐Ig), were applied at a 1:100 dilution and incubated overnight at 4°C. After washing, sections were incubated with fluorescently labeled secondary antibodies for 1 h, followed by counterstaining with DAPI for 5 min. For immunofluorescence and TUNEL analyses, three rats per group were randomly selected. Three to five fields were analyzed per rat, and the mean value of these fields was used as the value for each biological replicate. Data are presented as the mean ± standard deviation (SD).

### 2.7. TUNEL Assay

Apoptotic cells were detected using the TUNEL Apoptosis Detection Kit (Beyotime Biotechnology, C1086) according to the manufacturer’s protocol. Tissue sections were fixed in 4% paraformaldehyde (PFA) for 20 min, permeabilized with 0.1% Triton X‐100 for 10 min, and incubated with the TUNEL reaction mixture for 1 h at 37°C. Negative controls omitted the TdT enzyme. Samples were counterstained with DAPI and visualized under a fluorescence microscope (Olympus, Japan). For TUNEL quantification, three random fields within the condylar cartilage were analyzed for each rat, and the mean value of these fields was used as one biological replicate.

### 2.8. Statistical Analysis

Statistical analyses were performed using GraphPad Prism 10.0. One‐way ANOVA followed by Tukey’s multiple comparison tests was used for comparisons among multiple groups at each time point. Two‐way ANOVA was used to assess the main effects of HFD and CUS and their interaction. A significant interaction term was interpreted as statistical evidence supporting an interaction between HFD and CUS. Data are expressed as the mean ± SD. A *p*‐value < 0.05 was considered statistically significant.

## 3. Results

### 3.1. Effects of HFD and CUS on Body Weight and Serum Biochemistry

**Figure 1:**
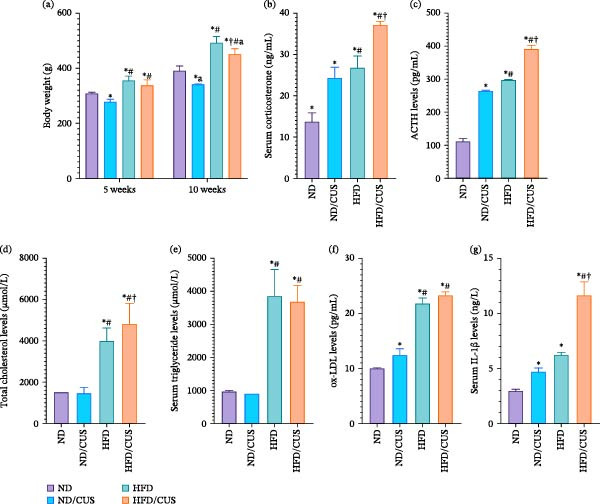
Changes of body weight and serum biochemical parameters. (a) Body weight. (b–g) Serum biochemical parameters were measured at 5 weeks after treatment. (b) Serum corticosterone. (c) Serum adrenocorticotropic hormone (ACTH). (d) Serum cholesterol. (e) Serum triglyceride. (f) Serum ox‐LDL. (g) Serum IL‐1β. ACTH, adrenocorticotropic; HFD, high‐fat diet; ND, normal diet; CUS, chronic unpredictable stress (*n* = 6;  ^∗^ *p* < 0.05 vs. ND group; #*p* < 0.05 vs. HFD group; † *p* < 0.05 vs. ND/CUS group; a *p* < 0.05 vs. the corresponding 5‐week group).

The HFD group showed a substantial increase in body weight, whereas the ND/CUS group showed slight weight reduction, and CUS partially blocked the HFD‐induced increase in weight in the HFD/CUS group, as compared to the ND group at 5 weeks posttreatment. These trends were consistently maintained at the 10‐week timepoint posttreatment (Figure 1a). At the 5‐week timepoint posttreatment, serum CORT and ACTH levels were significantly elevated in all ND/CUS, HFD, and HFD/CUS groups, with the highest levels observed in the HFD/CUS group, as compared to the ND group (Figure 1b,c). Serum levels of TC, triglycerides, and ox‐LDL were significantly increased in both the HFD and HFD/CUS groups compared with the ND and ND/CUS groups, with no significant difference between the HFD and HFD/CUS groups (Figure 1d–f). Serum IL‐1β levels were significantly increased in all ND/CUS, HFD, and HFD/CUS groups, with the highest levels in the HFD/CUS group, as compared to the ND group (Figure 1g). These results confirmed the effectiveness of the HFD and CUS protocols in rats.

### 3.2. HFD and CUS Synergistically Induced TMJOA‐Like Pathological Changes

**Figure 2:**
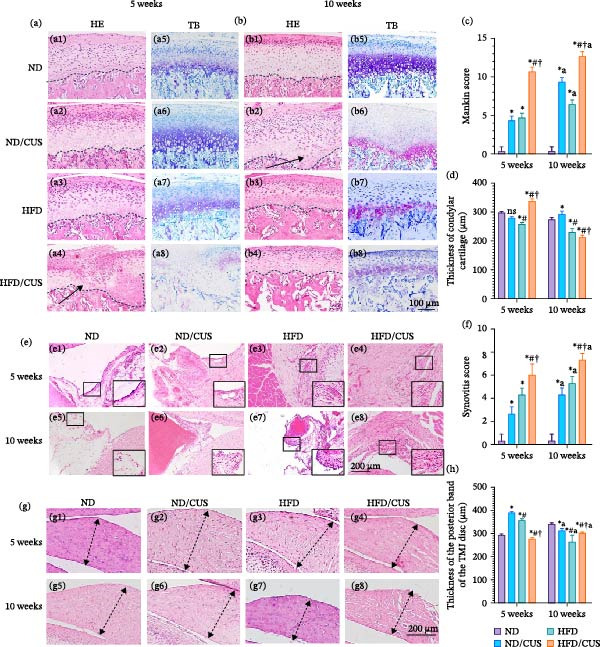
High‐fat diet and chronic unpredictable stress synergistically induced TMJOA‐like pathological changes. (a1–a4) HE‐stained condylar cartilage and (a5–a8) toluidine blue‐stained condylar cartilage from the ND, ND/CUS, HFD, and HFD/CUS groups, respectively, at 5 weeks; (b1–b4) HE‐stained condylar cartilage and (b5–b8) toluidine blue‐stained condylar cartilage from the same groups, respectively, at 10 weeks; (c, d) Quantification of Mankin scores and condylar cartilage thickness at 5 and 10 weeks; (e1–e8) HE‐stained synovial tissue from the ND, ND/CUS, HFD, and HFD/CUS groups at 5 and 10 weeks, respectively; (f) Quantification of synovitis scores at 5 and 10 weeks; and (g1–g8) HE‐stained posterior bands of the TMJ disc from the same groups at 5 and 10 weeks, respectively. (h) Quantification of posterior band thickness at 5 and 10 weeks. Data are presented as mean ± SD. Scale bars: 100 μm in panels a and b; 200 μm in panels e and g.  ^∗^ *p* < 0.05 vs. ND group; #*p* < 0.05 vs. ND/CUS group; †*p* < 0.05 vs. HFD group; a *p* < 0.05 vs. the corresponding 5‐week group; CUS, chronic unpredictable stress; HE, hematoxylin and eosin; HFD, high‐fat diet; ND, normal diet; SD, standard deviation; TB, toluidine blue staining; TMJ, temporomandibular joint; ns, not significant.

At 5 weeks posttreatment, HE staining revealed that the condylar cartilage only in the HFD/CUS group showed a marked increase in cartilage thickness, disruption of the fibrous cartilage layer, disorganization of chondrocyte arrangement and clustering of chondrocytes, and partial collapse of cartilage into the subchondral bone, while the HFD group or ND/CUS group only showed a marked cellular proliferation within the proliferative layer of the condylar cartilage, as compared to the ND group (Figure 2a,d). In addition, at 5 weeks posttreatment, TB staining showed a significant loss of ECM exclusively in the HFD/CUS group and mild ECM loss in the HFD and ND/CUS groups, as compared to the ND group (Figure 2a,h). At 10 weeks posttreatment, both HE and TB staining demonstrated a notable reduction in chondrocyte number and further loss of ECM in the HFD/CUS group (Figure 2b,d), whereas a marked increase in condylar cartilage thickness, accompanied by surface layer disruption, disorganization and clustering of chondrocytes, collapse of cartilage into the subchondral bone, and significant loss of ECM, was observed in the ND/CUS group. The HFD group also exhibited thinned condylar cartilage, along with a decrease in chondrocyte number and ECM loss, as compared to the ND group at 10 weeks posttreatment. (Figure 2b,h). The Mankin score was also highest in the HFD/CUS group among the four groups both at 5 weeks and 10 weeks posttreatment, while the HFD and ND/CUS groups also showed higher Mankin score than the ND group at 5 weeks posttreatment and higher at 10 weeks posttreatment, as compared to the ND group (Figure 2c).

At 5 weeks posttreatment, HE staining revealed that the synovial tissue in the HFD/CUS group exhibited significant fibroblast hyperplasia, thickening of the lining layer, and inflammatory cell infiltration in the sublining tissue, whereas the HFD and ND/CUS groups also showed marked cellular proliferation and mild inflammatory cell infiltration in the synovial lining layer, as compared to the ND group (Figure 2e). At 10 weeks posttreatment, HE staining revealed that the HFD/CUS group exhibited diffuse and severe synovial hyperplasia, tissue fibrosis, and massive inflammatory cell infiltration, while severe synovial hyperplasia was also observed in the ND/CUS group; and in the HFD group, significant fibroblast proliferation and vascular congestion were also observed, as compared to the ND group. Similarly, at 5 weeks or 10 weeks posttreatment, the synovitis score was the highest in the HFD/CUS group among the four groups, and the synovitis scores in the HFD and ND/CUS groups were also elevated at 5 weeks posttreatment and were further increased at 10 weeks posttreatment (Figure 2f).

At 5 weeks posttreatment, HE staining showed a significant increase in thickness of the posterior band of the TMJ disc in ND/CUS and HFD groups, whereas the HFD/CUS group unexpectedly exhibited a slight decrease in thickness of the posterior band, accompanied by a disordered fibrous structure. At 10 weeks posttreatment, a decrease in thickness of the posterior band was observed in all ND/CUS, HFD, and HFD/CUS groups, as compared to the ND group. The posterior band of the HFD/CUS group showed significant structural degradation and disorganization, characterized by a highly disordered, sparse, and loose arrangement of collagen fibers (Figure 2g,h).

### 3.3. HFD and CUS Synergistically Induced TMJOA‐Like Changes With Micro‐CT Evaluation

**Figure 3:**
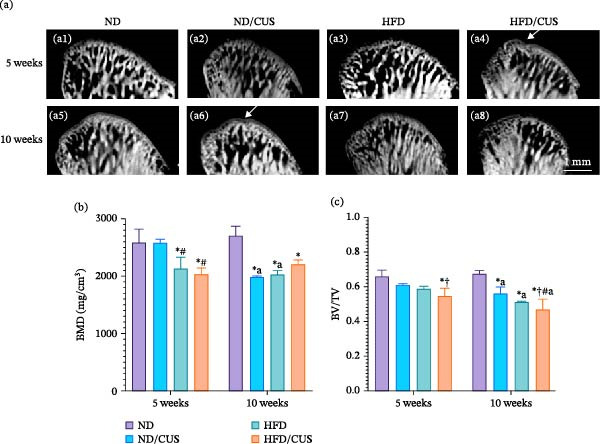
Micro‐CT analysis of condylar bone structure and quantitative assessment. (a), subpanels (a1–a4) show representative micro‐CT images of the condyles from the ND, ND/CUS, HFD, and HFD/CUS groups, respectively, at 5 weeks. Subpanels (a5–a8) show the corresponding groups, respectively, at 10 weeks. The white arrows in (a4, a6) indicate condylar defects. Please revise the figure legend accordingly. (b, c) bone structure‐related parameters BMD and BV/TV obtained from micro‐CT scanning. (Data are presented as mean ± SD. Scale bars: 1 mm. ^∗^ *p* < 0.05 vs. ND group; #*p* < 0.05 vs. ND/CUS group; †*p* < 0.05 vs. HFD group; a *p* < 0.05 vs. the corresponding 5‐week group; ns, not significant). BMD, bone mineral density; BV/TV, bone volume/tissue volume.

At 5 weeks posttreatment, 2D micro‐CT images revealed bone surface erosion and cortical bone discontinuity of the condyles only in the HFD/CUS group, as compared to the ND group (Figure 3a); quantitative analysis of bone parameters showed a significant decrease in BMD and BV/TV in the HFD/CUS group and also a slight decrease in BMD in the HFD group, as compared to the ND group (Figure 3b). At 10 weeks posttreatment, the HFD/CUS group continued to exhibit bone surface destruction, while the ND/CUS group showed significant erosive defects on the condylar bone surface and the HFD group showed a sparse trabecular bone structure. Although the three experimental groups showed comparable decreases in BMD, the HFD/CUS group still showed the greatest decrease in BV/TV among the experimental groups compared with the ND group (Figure 3b,c).

### 3.4. HFD and CUS Synergistically Promoted Chondrocyte Apoptosis in Condylar Cartilage

**Figure 4:**
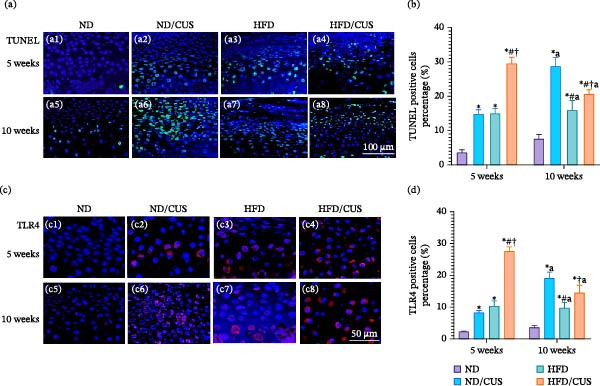
High‐fat diet and chronic unpredictable stress synergistically promoted chondrocyte apoptosis and TLR4 expression in TMJ condylar cartilage. (a), subpanels (a1–a4) show representative TUNEL‐stained images of condylar cartilage from the ND, ND/CUS, HFD, and HFD/CUS groups, respectively, at 5 weeks; subpanels (a5–a8) show the corresponding groups, respectively, at 10 weeks. (b) Quantification of the percentage of TUNEL‐positive cells. (c), subpanels (c1–c4) show representative TLR4 immunofluorescence images from the ND, ND/CUS, HFD, and HFD/CUS groups, respectively, at 5 weeks; subpanels (c5–c8) show the corresponding groups, respectively, at 10 weeks. (d) Quantification of the percentage of TLR4‐positive cells. Data are presented as mean ± SD. *n* = 3 rats per group. Scale bars: 100 μm in panel a and 50 μm in panel c.  ^∗^ *p* < 0.05 vs. ND group; #*p* < 0.05 vs. ND/CUS group; †*p* < 0.05 vs. HFD group; a *p* < 0.05 vs. the corresponding 5‐week group. CUS, chronic unpredictable stress; HFD, high‐fat diet; ND, normal diet; SD, standard deviation; TLR4, Toll‐like receptor 4; TUNEL, terminal deoxynucleotidyl transferase dUTP nick‐end labeling.

At 5 weeks posttreatment, the three experimental groups showed a significant increase in the percentage of TUNEL‐positive cells in the condylar cartilage compared with the ND group, with the highest percentage observed in the HFD/CUS group (Figure 4a,b). At 10 weeks posttreatment, the ND/CUS group unexpectedly showed the highest percentage of TUNEL‐positive cells among the three experimental groups, and this percentage was higher than that observed in the same group at 5 weeks. Although the percentages of TUNEL‐positive cells in the HFD and HFD/CUS groups remained higher than those in the ND group, they were lower than their corresponding values at 5 weeks.

### 3.5. HFD and CUS Synergistically Promoted Expressions of TLR4 and IL‐1β and Nuclear Translocation of NF‐κB in Condylar Cartilage

At 5 weeks posttreatment, the three experimental groups showed a significant increase in the percentage of TLR4‐positive cells in the condylar cartilage compared with the ND group, with the highest percentage observed in the HFD/CUS group (Figure 4c,d). At 10 weeks posttreatment, the ND/CUS group showed the highest percentage of TLR4‐positive cells among the three experimental groups and a higher percentage than that observed in the same group at 5 weeks. Although the percentages in the HFD/CUS and HFD groups remained higher than those in the ND group, the percentage in the HFD/CUS group was significantly lower than its corresponding value at 5 weeks.

**Figure 5:**
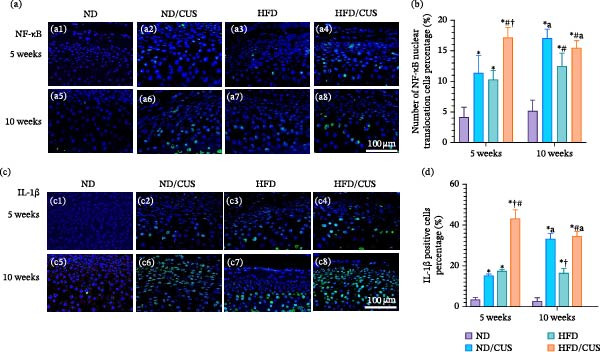
High‐fat diet and chronic unpredictable stress synergistically drove activation of the NF‐kB/IL‐1β signaling pathway in condylar cartilage. (a), subpanels (a1–a4) show representative NF‐κB p65 immunofluorescence images from the ND, ND/CUS, HFD, and HFD/CUS groups, respectively, at 5 weeks; subpanels (a5–a8) show the corresponding groups, respectively, at 10 weeks. (b) Quantification of the percentage of chondrocytes with nuclear NF‐kB p65 translocation. (c), subpanels (c1–c4) show representative IL‐1β immunofluorescence images from the ND, ND/CUS, HFD, and HFD/CUS groups, respectively, at 5 weeks; subpanels (c5–c8) show the corresponding groups, respectively, at 10 weeks. (d) Quantification of the percentage of IL‐1β‐positive cells. Data are presented as mean ± SD. *n* = 3 rats per group. Scale bars: 100 μm.  ^∗^ *p* < 0.05 vs. ND group; #*p* < 0.05 vs. ND/CUS group; †*p* < 0.05 vs. HFD group; a *p* < 0.05 vs. the corresponding 5‐week group. CUS, chronic unpredictable stress; HFD, high‐fat diet; IL‐1β, interleukin‐1 beta; ND, normal diet; NF‐kB, nuclear factor kappa B; SD, standard deviation.

At 5 weeks posttreatment, the three experimental groups showed an increase in the percentage of nuclear NF‐κB‐positive cells in the condylar cartilage as compared to the ND group, with the significantly highest percentage observed in the HFD/CUS group (Figure 5a,b). At 10 weeks posttreatment, the three experimental groups still showed a higher percentage of nuclear NF‐κB‐positive cells in the condylar cartilage as compared to the ND group, with a higher percentage observed in the HFD/CUS and ND/CUS groups than that in the HFD group and a comparable count for the HFD/CUS and ND/CUS groups, and with a higher percentage observed in the ND/CUS group, or with a lower count in HFD/CUS, or with no change in count in the HFD group, than that itself at 5 weeks posttreatment, respectively.

At 5 weeks posttreatment, the three experimental groups showed a significant increase in IL‐1β‐positive cell percentage observed in the condylar cartilage as compared to the ND group, with the significantly highest count in the HFD/CUS group (Figure 5c,d). At 10 weeks posttreatment, the three experimental groups still showed a higher IL‐1β‐positive cell count in the condylar cartilage as compared to the ND group, with a higher count in the HFD/CUS and ND/CUS groups than that in the HFD group and a comparable count for the HFD/CUS and ND/CUS groups, and with a higher count in the ND/CUS group, or with a lower count in HFD/CUS, or with no change in count in the HFD group, than that itself at 5 weeks posttreatment, respectively.

### 3.6. TLR4 Inhibitor TAK242 Partially Attenuated HFD/CUS‐Induced TMJOA‐Like Pathological Changes

**Figure 6:**
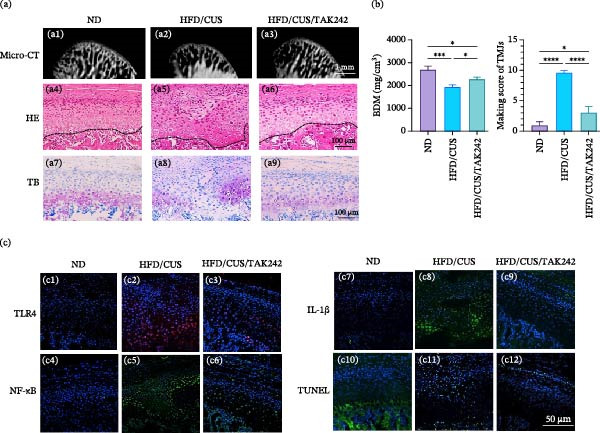
TAK242 alleviated HFD/CUS‐induced TMJOA‐like pathological changes and inhibited TLR4/NF‐kB/IL‐1β signaling. (a), subpanels (a1–a3) show representative micro‐CT images, (a4–a6) show representative HE‐stained images, and (a7–a9) show representative toluidine blue‐stained images of mandibular condyles from the ND, HFD/CUS, and HFD/CUS/TAK242 groups, respectively. (b) Quantitative analysis of BMD and Mankin scores. (c), subpanels (c1–c3) show representative TLR4 immunofluorescence images, (c4–c6) show representative NF‐κB p65 immunofluorescence images, (c7–c9) show representative IL‐1β immunofluorescence images, and (c10–c12) show representative TUNEL‐stained images of condylar cartilage from the ND, HFD/CUS, and HFD/CUS/TAK242 groups, respectively. Data are presented as mean ± SD. *n* = 6 rats per group. Scale bars: 1 mm for micro‐CT images, 100 μm for HE and toluidine blue staining, and 50 μm for immunofluorescence images.  ^∗^ *p* < 0.05,  ^∗∗∗^ *p* < 0.001,  ^∗∗∗∗^ *p* < 0.0001. BMD, bone mineral density; CUS, chronic unpredictable stress; HE, hematoxylin and eosin; HFD, high‐fat diet; IL‐1β, interleukin‐1 beta; ND, normal diet; NF‐kB, nuclear factor kappa B; SD, standard deviation; TAK242, TLR4 inhibitor; TB, toluidine blue staining; TLR4, Toll‐like receptor 4; TMJOA, temporomandibular joint osteoarthritis; TUNEL, terminal deoxynucleotidyl transferase dUTP nick‐end labeling.

The subchondral bone destruction, cartilage degradation, and proteoglycan loss were alleviated in the HFD/CUS/TAK242 group compared with the HFD/CUS group (Figure 6a). The increase in Mankin scores and the decrease in BMD were also partially reversed by TAK242 treatment (Figure 6b). Furthermore, the increases in TLR4, NF‐kB p65‐nuclear translocation, IL‐1β expression, and chondrocyte apoptosis were suppressed in the condylar cartilage of the HFD/CUS/TAK242 group compared with those of the HFD/CUS group (Figure 6c).

## 4. Discussion

The main findings of this study were that the combination of HFD and CUS synergistically induced TMJOA‐like pathological changes, especially significantly accelerating the onset and severity of OA‐like pathological changes more than HFD or CUS alone. We also provided preliminary evidence that these synergistic effects could potentially be mediated through the activation of the TLR4/NF‐κB/IL‐1β signaling pathway. To the best of our knowledge, our study provides experimental evidence for the potential interplay between lifestyle factors, HFD, and psychological stress in the pathogenesis of TMJOA.

The combination of HFD and CUS synergistically induced TMJOA‐like pathological changes, demonstrating multifaceted evidence from histopathology, radiology, serology, and molecular analyses. First, the combination of HFD‐ and CUS‐induced early‐stage TMJOA‐like pathological changes, including disruption of the fibrous cartilage layer, hyperplasia of the proliferative layer, disorganization of chondrocyte arrangement and cellular clustering, increased chondrocyte apoptosis, and reduced subchondral bone density in the HFD/CUS group within 5 weeks posttreatment, whereas only very slight pathological changes were observed in the ND/CUS group and HFD group. Second, at 10 weeks posttreatment, the combination of HFD‐ and CUS‐induced late‐stage TMJOA‐like pathological changes, characterized by severe cartilage matrix loss, depleted chondrocyte population, and further decrease in the BV/TV ratio of condylar bone, whereas the pathological changes in the ND/CUS group or HFD group were either still not comparable or at most comparable to those in the HFD/CUS group at 5 weeks posttreatment. The dynamic changes in the condylar proliferative layer were especially significant, from obvious hyperplasia in the proliferative layer at 5 weeks posttreatment to obvious reduction in the proliferative layer and in the full‐thickness cartilage at 10 weeks posttreatment in the HFD/CUS group. This biphasic change reflects a transition from early compensatory repair to late‐stage functional failure. While the initial hyperplasia acts as a compensatory response to the dual stressors, our TUNEL results indicate that prolonged HFD/CUS drives progressive chondrocyte apoptosis, ultimately exhausting this repair capacity and causing severe cartilage depletion by week 10. Third, the serum level of IL‐1β, synovitis score, and the number of cells with positive staining of TUNEL, TLR4, nuclear NF‐κB, and IL‐1β were also significantly higher in the HFD/CUS group at 5 weeks posttreatment. These findings suggested that HFD and CUS could potentially synergistically interplay in the pathogenesis of TMJOA. Our experimental design for observational time points at 5 or 10 weeks for the HFD/CUS group also reflected the early stage of TMJOA, characterized by proliferative changes, and the late stage of TMJOA, characterized by degenerative changes, closely in agreement with the clinical pathological changes of TMJOA [36, 37]. The clinical relevance of our results could be that avoidance of the interplay of CUS with HFD might be important in the prevention or treatment of TMJOA. These findings suggest that dietary modulation, including low‐fat or anti‐inflammatory dietary strategies, may warrant further investigation as a potential adjunctive approach for individuals with TMJOA, particularly those exposed to chronic stress [38].

HFD or CUS alone could also induce some TMJOA‐like pathological changes when the observational time was long enough. Our histopathological and micro‐CT analyses also showed that HFD or CUS alone showed a significant reduction in layer thickness and a disorganized chondrocyte arrangement in the condylar cartilage of the HFD or ND/CUS group at 10 weeks posttreatment. These changes are consistent with previous reports on HFD‐ or CUS‐induced pathological changes in the TMJ [39, 40]. However, the severity of the pathological changes as evaluated by the Mankin score in the HFD group or ND/CUS group was still mild as compared with that induced by the combination of the two factors even at 10 weeks posttreatment. Nevertheless, our results of HFD and CUS supported again that HFD or CUS alone was also a chronic, systemic risk factor for TMJOA.

The TMJ discs featured an increase or decrease in thickness in the early stage or late stage of TMJOA, respectively, in our animal models. We noticed that at 5 weeks posttreatment, the thickness of the posterior band was significantly increased in the HFD or ND/CUS group, but unexpectedly, the thickness already slightly decreased, accompanied by disorganization of collagen fibers in the HFD/CUS group, and that at 10 weeks posttreatment, the thickness of the posterior band was all decreased, accompanied by disorganization of collagen fibers. These results implied that the discs responded to the treatments with either an increase or decrease in posterior band thickness depending on the disease stage.

Considering that at 5 weeks posttreatment, the HFD/CUS group had already significant pathological changes in the condylar cartilage, the reason for the decrease in the thickness of the posterior band of discs in the HFD/CUS group at 5 weeks posttreatment would possibly be that the response of discs to the combination of HFD and CUS had already entered the thinning stage, that is, the stage for increase in the thickness of the posterior band had already occurred and been missed to be demonstrated due to the sample‐taking time point. This speculation could be proven in future experiments if the samples were taken as early as 3 weeks posttreatment for the HFD/CUS group. These observations raise the possibility that disc remodeling may occur early during disease progression and may contribute to subsequent condylar cartilage degeneration. This implication is consistent with the clinical observation, in which remodeling and thickening of the posterior band precede TMJ disc displacement and bone change [41, 42]. Biomechanically, the early thickening of the posterior band alters TMJ stress distribution and generates repetitive microtrauma for both the disc and condyle during condylar movements. This continuous microtrauma likely accelerates the subsequent destruction of the underlying cartilage and subchondral bone of the condyle and also further degenerative changes of the disc, leading to thinning of the posterior band.

The molecular mechanism underlying the induction of TMJOA‐like changes by the combination of CUS and HFD could be potentially mediated by the TLR4/NF‐κB/IL‐1β signaling pathway. At 5 weeks posttreatment, the number of cells with positive staining of TLR4, nuclear NF‐κB, and IL‐1β was all highest in the condylar cartilage in the HFD/CUS group, whereas the numbers of corresponding positive cells were also mildly increased in the HFD or CUS group, suggesting that TLR4 may be one molecular candidate involved in mediating the interplay between CUS and HFD. Importantly, the TAK242 experiment further supported the functional involvement of this pathway. TAK242 treatment partially reversed the HFD/CUS‐induced decrease in BMD, increase in the Mankin score, chondrocyte apoptosis, and activation of NF‐κB/IL‐1β signaling. Therefore, TLR4 signaling appears to functionally contribute to the inflammatory and degenerative changes induced by combined exposure to HFD and CUS. A previous study had already shown that TLR4 is upstream of NF‐κB in vascular smooth muscle cells [33] and that there exists a crosstalk between the HPA axis and TLR4 signaling [24–26]. Although we could not fully reproduce CUS in primary chondrocyte culture, the in vivo TAK242 inhibition experiment provides further evidence supporting the involvement of the TLR4/NF‐κB/IL‐1β axis in our model. Taken together, these findings suggest that activation of the TLR4/NF‐κB/IL‐1β signaling pathway may mediate, at least in part, the synergistic effects of combined CUS and HFD exposure on TMJOA‐like changes.

Our animal model of TMJOA could more accurately mimic the multifactorial etiology of TMJOA. Prevailing animal models of TMJOA often rely on singular, localized insults—such as surgically induced disc displacement [43], intraarticular chemical injections [3], partial disc discectomy [44], or artificial malocclusion [45]. The combination of chronic stress and a HFD amplified both systemic and local inflammatory states. Our serological data revealed that CUS significantly activated the HPA axis, manifested by elevated CORT levels—a response consistent with physiological stress in humans. Concurrently, HFD‐induced dyslipidemia and a body weight increase. We also noticed that CUS could decrease body weight, which is consistent with a previous study [46]. Critically, serum IL‐1β levels exhibited synergistic and maximal elevation in the HFD/CUS group, whereas the increase was substantially more modest with either HFD or CUS alone. Given the pivotal role of IL‐1β in the pathogenesis of OA [47, 48], this systemic increase in IL‐1β could be a key contributor to TMJOA development. The combination of these two factors fosters a potent proinflammatory systemic milieu. Specifically, HFD‐induced “meta‐inflammation” could produce more systemic proinflammatory cytokines, which could circulate into the TMJ microenvironment subsequently to enhance the degenerative changes of the disc and condyle in the CUS/HFD group. The elevated local IL‐1β signal in the disc and cartilage may be derived, at least in part, from chondrocytes as well as other resident or infiltrating inflammatory cells within the TMJ microenvironment. Therefore, our model of TMJOA provides a novel and more clinically relevant paradigm for investigating how these ubiquitous factors converge to drive the TMJOA pathology.

In conclusion, our study demonstrated that the combination of HFD and CUS synergistically induced TMJOA‐like pathological changes potentially through activation of the TLR4/NF‐κB/IL‐1β signaling pathway. These findings suggest that dietary modulation may be worth further investigation as a potential preventive or adjunctive strategy for TMJOA, particularly in individuals exposed to chronic stress.

## Funding

The study was supported by the National Natural Science Foundation of China (Grant 82170981).

## Conflicts of Interest

The authors declare no conflicts of interest.

## Data Availability

Data are contained within the article.
